# Biocompatible Properties and Mineralization Potential of Premixed Calcium Silicate-Based Cements and Fast-Set Calcium Silicate-Based Cements on Human Bone Marrow-Derived Mesenchymal Stem Cells

**DOI:** 10.3390/ma15217595

**Published:** 2022-10-28

**Authors:** Yemi Kim, Donghee Lee, Minjoo Kye, Yun-Jae Ha, Sin-Young Kim

**Affiliations:** 1 Department of Conservative Dentistry, College of Medicine, Ewha Womans University, Seoul 07986, Korea; 2 Department of Dentistry, College of Medicine, The Catholic University of Korea, Seoul 06591, Korea; 3 Department of Conservative Dentistry, Seoul St. Mary’s Hospital, College of Medicine, The Catholic University of Korea, Seoul 06591, Korea

**Keywords:** calcium silicate-based cement, human bone marrow-derived mesenchymal stem cell, biocompatibility, mineralization potential

## Abstract

Premixed calcium silicate-based cements (CSCs) and fast-set CSCs were developed for the convenience of retrograde filling during endodontic microsurgery. The aim of this study was to analyze the biocompatible properties and mineralization potential of premixed CSCs, such as Endocem MTA Premixed (EM Premixed) and EndoSequence BC RRM putty (EndoSequence), and fast-set RetroMTA on human bone marrow-derived mesenchymal stem cells (BMSCs) compared to ProRoot MTA. Using CCK-8, a significantly higher proliferation of BMSCs occurred only in the EM Premixed group on days 2 and 4 (*p* < 0.05). On day 6, the ProRoot MTA group had significantly higher cell proliferation than the control group (*p* < 0.05). Regardless of the experimental materials, all groups had complete cell migration by day 4. Alizarin Red-S staining and alkaline phosphatase assay demonstrated higher mineralization potential of all CSCs similar to ProRoot MTA (*p* < 0.05). The EndoSequence group showed more upregulation of *SMAD1* and *OSX* gene expression than the other experimental groups (*p* < 0.05), and all experimental cements upregulated osteogenic gene expression more than the control group (*p* < 0.05). Therefore, using premixed CSCs and fast-set CSCs as retrograde filling cements may facilitate satisfactory biological responses and comparable osteogenic potential to ProRoot MTA.

## 1. Introduction

Mineral trioxide aggregate (MTA) is conventionally used in endodontic microsurgery because of its biocompatibility and sealing ability [1]. ProRoot MTA (Dentsply Tulsa Dental Specialties, Tulsa, OK, USA) is considered one of the favorable prognostic retrograde filling materials and influences the proliferation of human bone marrow-derived mesenchymal stem cells (hBMSCs) and regeneration of destroyed apical bone [2]. However, due to major drawbacks of ProRoot MTA, such as difficulty handling and long setting time, various calcium silicate-based cements (CSCs) were developed [3,4,5,6]. In the context of bleeding, such as apical microsurgery, a more convenient material for the operator is favorable. In particular, premixed putty-type materials are easier to apply during surgical procedures.

Premixed CSCs are suggested to replace conventional powder liquid-type CSCs, reducing the influence of mixing ratio and inconvenience of cement delivery [7,8,9]. EndoSequence BC RRM putty (EndoSequence; Brasseler Co., Savannah, GA, USA) is prepared as putty, easy to apply for clinical use, and reported to be a bioactive material [8]. EndoSequence uses glycerol instead of water and does not harden in its package, but sets after being exposed to a wet condition; it contains tantalum oxide as a radiopacifying agent [10]. Many previous studies comparing ProRoot MTA and EndoSequence found no meaningful differences in the success rate of apical microsurgery according to the material [11,12,13,14]. Both materials had a high success rate because both have biocompatibility and osteogenic potential. Calcium phosphate (CaP) components in hydraulic CSCs markedly modified the chemical–physical properties, improving the cell response and increasing the apatite-forming ability.

Endocem MTA Premixed (EM Premixed; Maruchi, Wonju, USA) was also developed to overcome the mixing inconvenience of ProRoot MTA. It is an injectable syringe-type CSC that can be applied easily on the retrograde preparation area or root resorption area [15]. In a previous study, EM Premixed showed favorable biocompatibility and osteogenic potential on bone marrow stem cells (BMSCs) similar to ProRoot MTA [16]. RetroMTA (BioMTA, Seoul, Korea) has a fast setting time and favorable histological response in cases of partial pulpotomy of human permanent teeth [17,18]. It is also provided in a powder and liquid form, but it is easily applicable because the initial setting time of RetroMTA is faster than that of ProRoot MTA. In a previous study, RetroMTA was reported to have similar angiogenic effects and biological responses as ProRoot MTA [19,20].

The aim of this study was to compare the biological responses and mineralization activity on hBMSCs of EndoSequence, EM Premixed, and RetroMTA to ProRoot MTA.

## 2. Materials and Methods

### 2.1. Human Bone Marrow-Derived Stem Cell

This research was approved by the IRB of Seoul St. Mary’s Hospital (IRB No. MC20SESI0067). The hBMSCs (Catholic MASTER Cells) were prepared from the Catholic Institute of Cell Therapy (CIC, Seoul, Korea) as described previously [16]. In a colony forming test, most hBMSCs revealed a fusiform similar to other types of mesenchymal stem cells. Entire experimental methods were processed in sterile environments.

### 2.2. Experimental Disks of Retrograde Filling Cements

The cements tested in this study were EndoSequence, EM Premixed, RetroMTA, and ProRoot MTA. The components of these materials are presented in Table 1. All cements were prepared according to the manufacturer’s instructions. Experimental disks were made with a diameter of 6 mm and height of 3 mm using a sterile rubber mold. All specimens were sterilized for 4 h in a clean bench using ultraviolet light during setting and stored in an incubator at 100% humidity and 37 °C for 72 h.

### 2.3. Cell Viability

**Figure 1:**
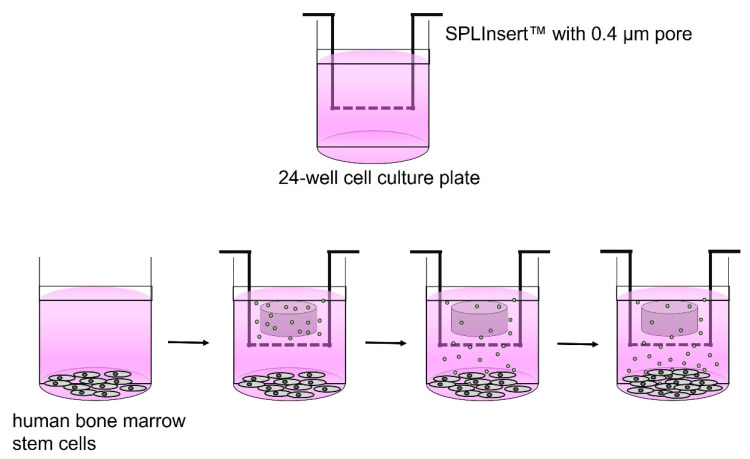
Experimental procedures of cell viability, cell migration, and alkaline phosphate activity assays.

The Cell Counting Kit (CCK-8) test (CK04-13; Dojindo, Kumamoto, Japan) was used to measure the cytotoxicity of four kinds of retrograde filling cement disks. The proliferation of hBMSCs was analyzed 2, 4, and 6 days after incubation. The hBMSCs were cultured at a density of 1.0 × 10⁴ cells/well on 24-well cell culture plates (SPL Life Sciences, Pocheon, Korea) including growth medium. After 24 h of cell adhesion, each disk was placed individually into an insert with 0.4-μm pore (SPLInsert; SPL Life Sciences), and the insert was placed over the attached cells. The optical density was measured after 2, 4, and 6 days after insertion of experimental disks (Figure 1). More detailed methods were described previously [21,22]. Each group was measured in octuplicate.

### 2.4. Cell Migration Assay

The wound healing test was used to measure cell migration. The hBMSCs were plated with density of 3.5 × 10⁴ cells/well on 24-well cell culture plates with growth medium. After 24 h of cell adhesion, a 1000 μL pipette tip was used to make scratches in the center of the binding cell layer. Cell migration was measured after 1, 2, 3 and 4 days of hBMSC incubation using a phase contrast microscope (Olympus, Tokyo, Japan). We used ImageJ software (ver. 1.53a; National Institutes of Health, Bethesda, MD, USA) for measuring the surface area covered by cells. More detailed methods were described previously [22]. Each group was measured in quadruplicate.

### 2.5. Alkaline Phosphatase Activity

The mineralization potential of hBMSCs was evaluated after 3 or 6 days of incubation using the alkaline phosphatase (ALP) assay. The hBMSCs were plated at a density of 3.5 × 10⁴ cells/well on 24-well cell culture plates with osteogenic medium, and an experimental disk was placed on each insert. On days 3 and 6, attached cells were dissolved with Triton X-100 (AnaSpec, Fremont, CA, USA) and cultured at 37 °C for 15 min. For ALP analysis, a total of 50 μL p-nitrophenyl phosphate (pNPP; AnaSpec) was added to the upper layer of each sample and shaken lightly for 30 s to mix the reagent. For visualization, cells were fixed with 4% paraformaldehyde for 1 min and processed with alkaline phosphatase detection kit (EMD Millipore Corp., Billerica, MA, USA). More detailed methods were described previously [16]. Each group was measured in sextuplicate.

### 2.6. Alizarin Red-S Staining Assay

Calcified nodule formation ability of hBMSC was evaluated after 7 and 14 days of incubation using Alizarin Red-S (ARS) staining assay. After production of all disks, we measured the weight of each disk, adjusted the osteogenic medium volume according to the weight, and produced eluate stock as a 50 mg/mL concentration. In order to release disk components into the osteogenic medium, all disks with media were stored in an incubator at 100% humidity and 37 °C for 7 days. Supernatant of each eluate was filtered using a 0.2 μm membrane filter, diluted 1/10, and prepared to a final concentration of 5 mg/mL.

The hBMSCs were sprayed at a density of 2.0 × 10⁴ cells/well and incubated for 14 days using each retrograde filling cement eluate. On days 7 and 14, the cells were treated with 2% ARS solution (ScienCell, Carlsbad, CA, USA) for 20 min and stained with 10% cetylpyridinium chromide (Sigma-Aldrich, St. Louis, MO, USA) for 15 min. Each group was measured in sextuplicate.

### 2.7. Quantitative Real-Time Polymerase Chain Reaction

The hBMSCs using each retrograde filling cement eluate were incubated for 9 days to measure osteocalcin (*OCN*), mothers against decapentaplegic homolog 1 (*SMAD1*), and osterix (*OSX*), and 14 days to measure dentin matrix protein-1 (*DMP-1*) and dentin sialophosphoprotein (*DSPP*). RNA isolation and real-time polymerase chain reactions (RT-PCR) were performed according to our previous study [21]. Primers were designed using GenBank (Table 2). The mRNA levels were normalized to GAPDH and expressed as the fold change.

### 2.8. Statistical Analysis

The SPSS software program (ver. 24.0; IBM Corp., Armonk, NY, USA) was used for statistical analyses. We confirmed the data normality first and performed a repeated measures analysis of variance (RM ANOVA) for overall experiments. If the significance between experimental groups was confirmed after RM ANOVA, we performed one-way analysis of variance (one-way ANOVA) and Tukey post-hoc test. *p* < 0.05 was considered significant.

## 3. Results

### 3.1. Cell Viability 

**Figure 2:**
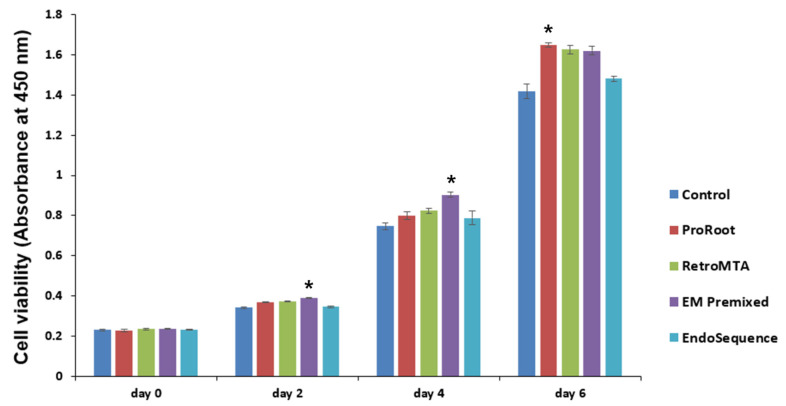
Comparison of cell viability by experimental cement. * *p* < 0.05 compared to the control group at each time point. Error bars mean standard deviations.

Significantly higher cell proliferation was observed only in the EM Premixed group on days 2 and 4 (Figure 2, *p* < 0.05) compared to the control group. On day 6, the ProRoot MTA group had significantly higher cell proliferation than the control groups (Figure 2, *p* < 0.05). EndoSequence resulted in no significant differences compared to the control group in any experimental period.

### 3.2. Cell Migration Assay

**Figure 3:**
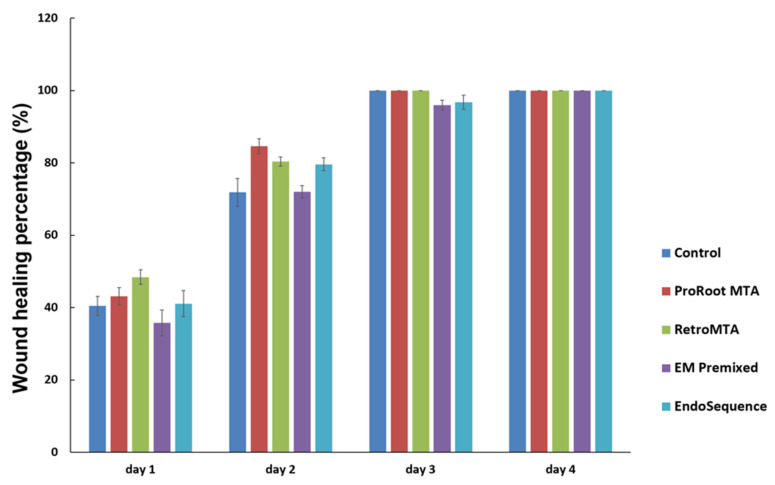
Comparison of cell migration by experimental cement. There were no significant differences between the experimental and control groups at each time point. Error bars mean standard deviations.

**Figure 4:**
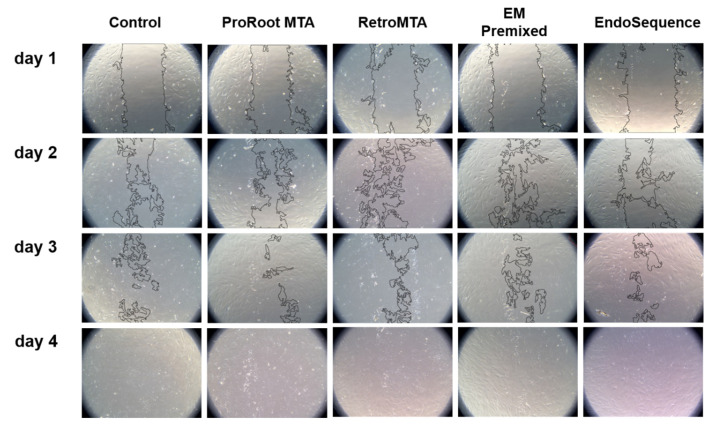
Representative images of cell migration assay. Black lines indicated that cells were migrated from both sides into the scratch area in the center.

Regardless of the experimental material, wound healing percentages meaningfully increased over time (Figure 3). On day 4, all experimental cements exhibited almost complete cell migration. Representative images of wound healing are shown in Figure 4. 

### 3.3. Alkaline Phosphatase Activity

**Figure 5:**
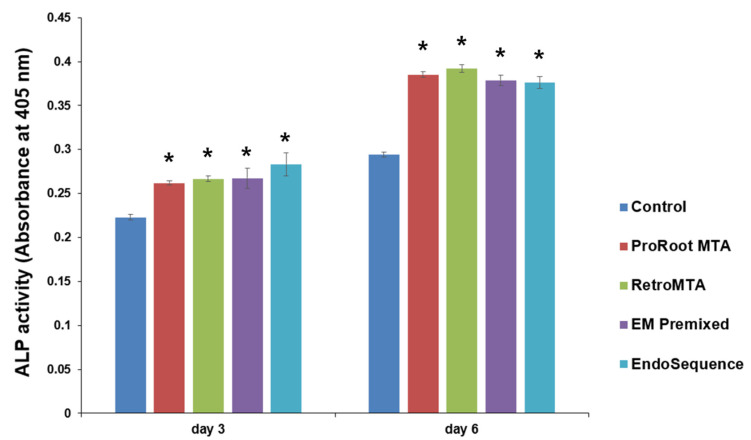
Comparison of alkaline phosphatase (ALP) activity by experimental cement. * *p* < 0.05 compared to the control group at each time point. Error bars mean standard deviations.

**Figure 6:**
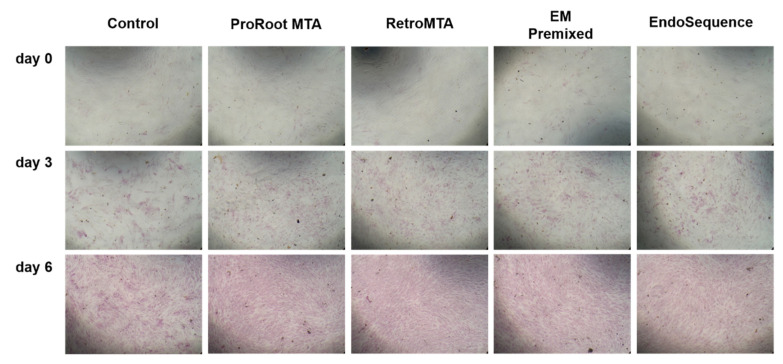
Representative images of alkaline phosphatase (ALP) activity.

On days 3 and 6, all experimental groups had higher ALP activity than the control group (Figure 5, *p* < 0.05), and no significant differences were found between experimental cements. All groups had higher ALP activity over time, and the density of ALP staining was increased on day 6 compared to day 3 (Figure 6).

### 3.4. Alizarin Red-S Staining Assay

**Figure 7:**
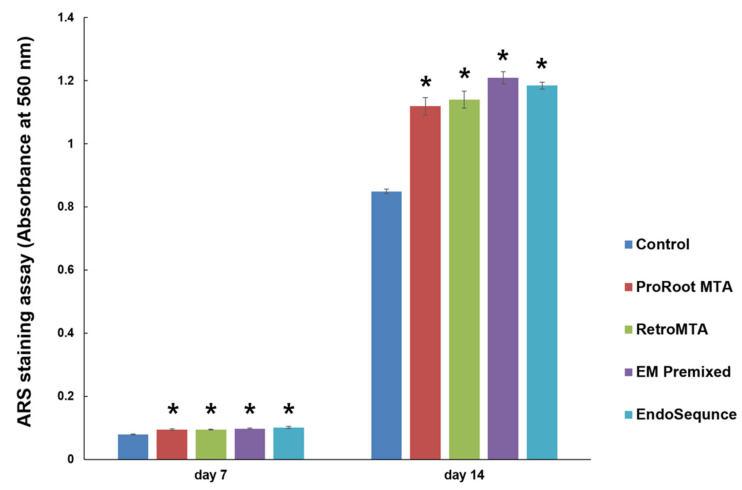
Comparison of Alizarin Red-S (ARS) staining by experimental cement. * *p* < 0.05 compared to the control group at each time point. Error bars mean standard deviations.

**Figure 8:**
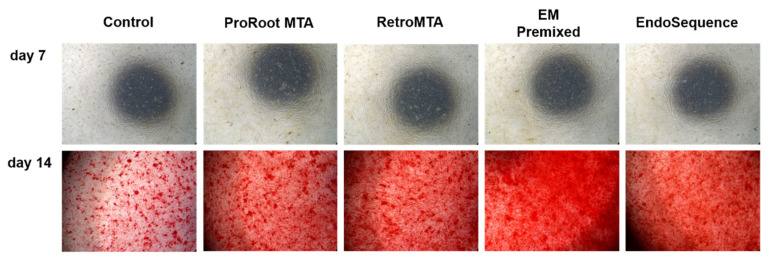
Representative images of Alizarin Red-S (ARS) staining.

Overall, the experimental groups had increasing calcium nodule density on day 14 compared to day 7 (Figure 7). We found no significant differences between experimental cements on days 7 and 14; however, all experimental groups had significantly higher ARS staining than the control group (Figure 7, *p* < 0.05). Representative images of ARS staining are shown in Figure 8.

### 3.5. Quantitative Real-Time Polymerase Chain Reaction

**Figure 9:**
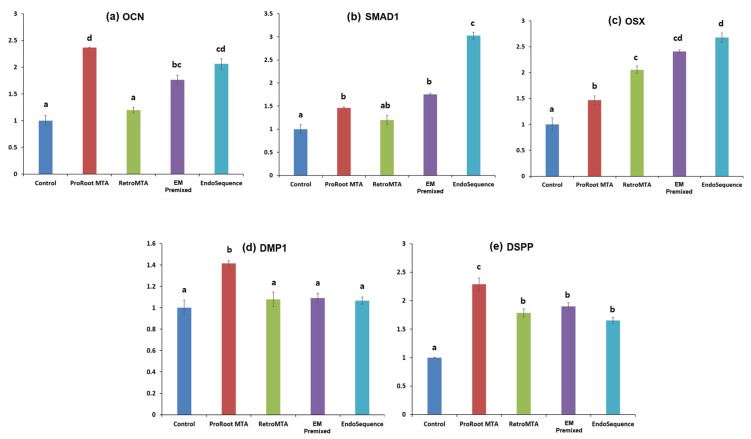
(**a**–**e**) mRNA expression of select genes. *OCN, SMAD1*, and *OSX* on day 9. *DMP-1* and *DSPP* on day 14. Different superscript letters mean statistically significant differences between experimental groups. Error bars mean standard deviations.

The ProRoot MTA group showed more upregulation of *OCN* gene expression on day 9 (Figure 9a, *p* < 0.05), and more upregulation of *DMP-1* and *DSPP* genes on day 14 than the other experimental groups (Figure 9d,e, *p* < 0.05). However, the EndoSequence group showed more upregulation of *SMAD1* and *OSX* genes expression on day 9 than the others (Figure 9b,c, *p* < 0.05). All experimental materials upregulated osteogenic gene expression more than the control group (Figure 9).

## 4. Discussion

In this study, EndoSequence, EM Premixed, and RetroMTA showed favorable biocompatibility and mineralization potential similar to ProRoot MTA. We used the CCK-8 and wound healing assays to evaluate biocompatibility. EM Premixed resulted in higher cell proliferation on days 2 and 4, whereas ProRoot MTA had higher cell proliferation on day 6. EM Premixed is a recently launched syringe-type bioceramic that is easy to use for the clinician, with a setting time shorter than that of ProRoot MTA. The composition of EM Premixed is almost similar to Endoseal TCS (Maruchi, Wonju, Korea). However, there are not enough published studies regarding EM Premixed; therefore, we included this material in our experimental groups. It is mostly comprised of zirconium dioxide (45–55%). The addition of metal oxides improves the mineralization activity [23]. Zirconium is a natural component occurring in the bone and verified to be a biocompatible osteoinductive material that can reinforce the proliferation and differentiation of osteoblasts [23]. In previous studies, EndoSequence had similar favorable results in biological evaluations compared to ProRoot MTA [24,25,26,27], which was confirmed in our study. In the CCK-8 analysis, EndoSequence showed no significant difference compared to ProRoot MTA on days 2 and 4. However, EndoSequence resulted in lower cell viability compared to the ProRoot MTA group on day 6. The reason for the lower cell viability was that the small particles from the EndoSequence disk passed through the insert onto the surface of the BMSCs attached during the CCK-8 analysis.

We used BMSCs instead of dental pulp stem cells (DPSCs) in this study. EndoSequence showed more favorable biocompatibility results in DPSCs than BMSCs [28]. In a previous study, the mitogenic efficacy on periodontal ligament stem cells and DPSCs was > 49% in the EndoSequence group compared to 26% in the ProRoot MTA group. However, the mitogenic efficacy of hBMSCs was similar between the EndoSequence and ProRoot MTA groups [28]. The main component of EndoSequence is tricalcium and dicalcium silicate, and it is premixed with zirconium oxide or tantalum oxide to increase the radiopacity of the material [10]. A previous study showed that tricalcium silicate-based biomaterials influence the intracellular Ca^2+^ dynamics, resulting in the cellular differentiation and mineralization potential of hDPSCs [29]. During the wound healing assay, meaningful differences in cell migration were not observed between experimental materials. On day 4, all scratches on the BMSC cultures were completely healed.

In this research, we used ALP activity, ARS staining, and qRT-PCR to evaluate the mineralization potential. The RetroMTA, EM Premixed, EndoSequence, and ProRoot MTA groups had higher ALP levels and ARS than the control group. The highest *OCN*, *DMP-1*, and *DSPP* expression was found in the ProRoot MTA group and the highest *SMAD1* and *OSX* expression in the EndoSequence group. *DMP-1* and *DPSS* genes are expressed in later stages compared to *SMAD1, OSX,* and *OCN.* Therefore, we evaluated the expression of *SMAD1, OSX,* and *OCN* genes on day 9, and *DMP-1* and *DPSS* genes on day 14.

In a previous study, EndoSequence showed upregulation of *ALP* and *DSPP* gene expression in stem cells of the apical papilla [30], whereas ProRoot MTA had a high expression of integrin binding sialoprotein (*IBSP*) and runt-related transcription factor 2 (*Runx2*), representing a critical role in osteoblastic differentiation. However, both ProRoot MTA and EndoSequence had high ARS staining [30], which suggested that both materials have a favorable mineralization potential.

OSX, a zinc finger-containing transcription factor expressed in all developing bones [31], acts downstream of *Runx2* and regulates osteoblast differentiation and proliferation [32]. A previous study by Sun et al. demonstrated upregulation of *OSX* gene expression in the EndoSequence putty group compared to the NeoPutty (NuSmile, Houston, TX, USA) and control groups [33]. This is a similar result as our study, in which more upregulation of *OSX* expression was found in EndoSequence than ProRoot MTA. Sun et al. also reported that *Runx2*, *DSPP*, and ALP expression were greater with EndoSequence than in the control group [33].

In a previous study, both EndoSequence putty and Biodentine (Septodont, Saint Maur des Fosses, France) resulted in higher cumulative calcium release potential than MM-MTA (MicroMega, Besançon, France) [34]. The ability to release calcium ions may be important for promoting carbonated apatite layer formation. Abu Zeid et al. found calcium phosphate precipitates on the surface of regular-set and fast-set EndoSequence surfaces [35]. This may be beneficial for improving the sealing ability of these calcium silicate-based materials.

OCN, which is a marker of late osteogenic differentiation, was expressed at higher levels in the ProRoot MTA, EndoSequence, and EM Premixed groups in this study. This is in agreement with the previous study by Oh et al. [36]. In their study, EndoSeal TCS resulted in a more significant upregulation of *OCN* and *Runx2* gene expression than other root canal sealer groups. The major component of EndoSeal TCS is tricalcium silicate, not dicalcium silicate; therefore, it can produce higher levels of calcium hydroxide than other root canal sealers [36]. Calcium hydroxide facilitated the phosphorylation of c-Jun N-terminal kinase (JNK), p38, and extracellular signal regulated kinase (ERK). These genes are implicated in calcium hydroxide-related cell migration, mineralization ability, and osteogenic potential [37]. Therefore, EndoSeal TCS can reveal high osteogenic potential. EM Premixed is known to have a similar composition as Endoseal TCS and, therefore, may have a favorable mineralization potential. In our previous study, EM Premixed exhibited more calcium nodule formation than the control group, and significant differences were not found between EM Premixed, ProRoot MTA, and RetroMTA on day 14 [16]. These results are similar to those of this study, but there are not enough studies regarding EM Premixed and more research regarding this material is necessary.

In this study, *OCN* expression was more significantly downregulatged in the RetroMTA group than the other experimental groups, but *SMAD1*, *OSX*, *DMP-1*, and *DSPP* expression was similar to the expression in the EM Premixed group. Bakhtiar et al. reported that 64% of the RetroMTA group had irregular hard tissue formation and 9% did not show any hard tissue formation, compared to 100% of the ProRoot MTA group having no inflammation [17]. In another study, RetroMTA resulted in a 50% complete calcific barrier compared to 69% with ProRoot MTA [18]. In their study, RetroMTA exhibited lower pulpal responses and smaller calcific barrier area compared to ProRoot MTA, but significant differences were not found between the two groups [18]. Taking these findings and ALP activity, ARS staining ability, and mRNA gene expression in this study into consideration, RetroMTA could be a favorable bioactive material similar to EndoSequence and EM Premixed.

We followed a previous guideline to report the results of this study [38]; however, some limitations have to be considered about the results of this study. First, we used BMSCs instead of DPSCs. Some CSCs were developed with the expectations of ideal cell proliferation and differentiation of DPSCs, and mineralization potentials of CSCs could be different according to various cell types. Second, experimental materials in this study were tested in set state. During apical microsurgery, we applied retrograde filling materials with unset state. Therefore, comparison between set and freshly mixed materials will be necessary. Third, physicochemical properties, such as solubility, dimensional stability, and radiopacity, were not evaluated in this study. Fourth, we evaluated only five genes out of many variable osteogenic markers. During osteoblastic differentiation of BMSCs, a complex network of intracellular signaling molecules, calcium ion release, and interactions of cells with the extracellular matrix play a key role in this differentiation process. Therefore, high accurate and reproducible identification of variable gene expression, such as RNA-sequencing [39] is recommended.

## 5. Conclusions

Conventional ProRoot MTA has good biocompatibility and bioactivity. To overcome the drawbacks of MTA during endodontic microsurgery, fast set CSCs, such as Retro MTA, and premixed CSCs, such as EndoSequence and EM Premixed, were developed. All CSCs demonstrated satisfactory biological responses and comparable osteogenic potential to ProRoot MTA. This study was performed in vitro. Therefore, to have confidence in various CSCs, in vivo study and randomized controlled clinical trials will be needed.

## Figures and Tables

**Table 1:** Manufacturer and chemical composition of material used in this study.

Material	Manufacturer	Components	Lot Number
ProRoot MTA	Dentsply Tulsa Dental Specialties, Tulsa, OK, USA	Portland cement (tricalcium silicate, dicalcium silicate, and tricalcium aluminate) 75% Calcium sulfate dehydrate (gypsum) 5% Bismuth oxide 20%	0000186484
RetroMTA	BioMTA, Seoul, Korea	Calcium carbonate 60–80% Silicon dioxide 5–15% Aluminum oxide 5–10% Calcium zirconia complex 20–30%	RM1810D14
Endocem MTA Premixed	Maruchi, Wonju, Korea	Zirconium Dioxide 45–55% Calcium silicate 20–25% Calcium aluminate 1–5% Calcium sulfate 1–5% Dimethyl sulfoxide 20–25% Thickening agent 1–5%	C2304160716
EndoSequence BC RRM putty	Brasseler Co., Savannah, GA, USA	Tricalcium and dicalcium silicate, calcium sulfate, tantalite, zirconia and proprietary organic liquid	1808BPP

**Table 2:** Primer sequences for evaluating osteogenic gene expression in hBMSCs.

Gene	Primer Sequence
Osteocalcin (*OCN*)	Forward 5’-GTG CAG AGT CCA GCA AAG GT-3′ Reverse 5′-TCA GCC AAC TCG TCA CAG TC-3′
Mothers against decapentaplegic homolog 1 (*SMAD1*)	Forward 5′-CCA CTG GAA TGC TGT GAG TTT CC-3′ Reverse 5′-GTA AGC TCA TAG ACT GTC TCA AAT CC-3′
Osterix (*OSX*)	Forward 5′-CCT GGC TGC GGC AAG GTG T-3′ Reverse 5′-GAT CTC CAG CAA GTT GCT CTG C-3′
Dentin matrix protein-1 (*DMP-1*)	Forward 5′-TGG TCC CAG CAG TGA GTC CA-3′ Reverse 5′-TGT GTG CGA GCT GTC CTC CT-3′
Dentin sialophosphoprotein (*DSPP*)	Forward 5′-GGG AAT ATT GAG GGC TGG AA-3′ Reverse 5′-TCA TTG TGA CCT GCA TCG CC-3′
GAPDH	Forward 5′-TGT CAT CAA CGG GAA GCC-3′ Reverse 5′-TTG TCA TGG ATG ACC TTG-3′

## Data Availability

The datasets used and/or analyzed during this study are available from the corresponding author on reasonable request.
